# Use, Misuse, and Complications of Contact Lens Among the General Population of the Kingdom of Saudi Arabia

**DOI:** 10.7759/cureus.51368

**Published:** 2023-12-30

**Authors:** Reem S AlSarhan, Bandar M Abuageelah, Ahmed A Alahmadi, Mona H Alfaifi, Kholoud M Alghamdi, Abdulrahman Alamri

**Affiliations:** 1 College of Medicine, Princess Nourah Bint Abdulrahman University, Riyadh, SAU; 2 Department of Medicine and Surgery, Batterjee Medical College, Jeddah, SAU; 3 Department of Medicine and Surgery, Umm Al-Qura University, Mecca, SAU; 4 Faculty of Medicine, Al-Baha University, Al-Baha, SAU; 5 Department of Ophthalmology, College of Medicine, King Khalid University, Abha, SAU

**Keywords:** psychological factors, knowledge levels, complications, usage patterns, contact lenses

## Abstract

Purpose: This study aims to assess contact lens usage patterns, complications, knowledge levels, and the impact of psychological factors on misuse among the Saudi population.

Methods: In this study, we distributed our questionnaire through multiple social media platforms among patients who are using cosmetic or medical contact lenses regardless of the purpose of wearing them. A total of 1,708 contact lens users participated.

Results: Most participants were female (86.4%) and aged 21-40 years (65.7%). The purpose for usage was cosmetic (36.1%) or medical (28.7%), with some using them for both purposes (35.1%). Complications have been faced, with 26.3% experiencing issues due to lens usage, 56.8% facing visual problems, and 45.8% sometimes feeling discomfort with contact lens usage. Allergic reactions were reported by 35.2%. Higher knowledge levels were associated with participants aged 21-40 years (p = 0.009), irregular ophthalmologist visits (p = 0.032), and cosmetic use (p = 0.027). Better practice patterns were observed among urban residents (p = 0.049), higher-income earners (p = 0.002), cosmetic users (p < 0.001), and those with fewer complications (p < 0.001). Psychological factors significantly influenced misuse, with 15.4% of participants indicating its impact. Notably, a subset of these participants (16.2%) perceived prolonged wear of contact lenses as a manifestation of personal insecurity.

Conclusions: Several sociodemographic factors, including place of residence, income, and difficulty, influence the use of contact lenses. It is also necessary to consider psychological issues such as low self-esteem and social acceptance while encouraging the safe use of contact lenses.

## Introduction

Contact lenses (CLs) are optical devices worn on the eye and put directly on the cornea's surface [1]. They come in various shapes and sizes, including hard, soft, semisoft, and gas-permeable lenses. CLs are used for cosmetic improvement, correcting refractive problems, and other therapeutic purposes [2]. They are generally safe when used appropriately. The advantages of CLs increase their popularity and demand globally, and as CLs' popularity and demand increase, use-related problems also increase [3]. Since CLs are easily obtained without a prescription, the public may need to know their hazards and benefits. For all age groups, including health professionals, the educational level about the safe use of CLs should be raised to include proper usage, cleanliness procedures, and potential risks of being misused [4,5]. Unfortunately, neglecting to follow medical recommendations may result in unexpected complications such as corneal abrasion, corneal ulcer, keratitis, dry eye, and conjunctivitis, all of which are frequent complications associated with the misuse of CLs [6,7]. The most severe complication is blindness induced by microbial keratitis and endophthalmitis [8]. Young users were unaware of these complications, and most favor contact lens usage despite the ocular complications for cosmetic reasons [9,10].

Even though many young people in Saudi Arabia use CLs, little research has examined their knowledge and usage of the lenses. This study analyzes the knowledge and associated complications of CL misuse among Saudi Arabia's population at the level of the kingdom's five major areas: the Western region, the Central region, the Southern region, the Eastern region, and the Northern region.

## Materials and methods

This cross-sectional online survey study evaluated Saudi people's knowledge of CL use, abuse, and problems. Saudi citizens and residents in Saudi Arabia's five main areas (the Central, Eastern, Western, Northern, and Southern regions) were given access to the survey's online questionnaire. The study included 1079 participants in total. The information was gathered between July 2 and July 13, 2023. Ethical approval for the study was granted by Princess Nourah Bint Abdulrahman University (approval No: 23-0525).

The inclusion criteria for the selection of subjects for this study included Saudi citizens who were ≥ 18 years old and wore medical or cosmetic lenses. No gender or geographic restrictions were enforced. The exclusion criteria included Saudi citizens under 18 and those with chronic debilitating diseases.

The questionnaire was created following the literature review and was modified from a prior study [11,12]. After reviewing the questionnaire's content, the committee of expert researchers (faculty members) decided if the instrument had been validated. The questionnaire contents were translated into Arabic back-to-back until they were understood and covered the study's goals. A Google Form (Google LLC, Mountain View, California, United States) with the complete questionnaire was uploaded, and an open link was created. 

The survey utilized a convenience sampling technique. The researchers initially sent a link to the survey via social media platforms (Twitter/X (X Corp., San Francisco, California, United States), WhatsApp (Meta Platforms, Inc., Menlo Park, California, United States), and Telegram (Telegram FZ LLC, Dubai, United Arab Emirates) to their primary contacts to ensure it was dispersed to different parts of Saudi Arabia. The survey was then forwarded to the primary contacts' connections, and so on.

The first part of the questionnaire included information about sociodemographic data. The second part contained participants' characteristic questions regarding their CL use. The third part consisted of the practice patterns among CL users regarding hygiene, accessory care, and the need to seek urgent and routine eye care for discomfort or acute eye complications they experienced. The fourth section of the questionnaire assessed the user's perception of CL. The final section of the survey contained questions regarding CL use and its association with psychological background. Answers such as "yes," "no," or "I do not know" were used to assess the responses to each knowledge question. 

The study assessed the participants' knowledge and practice regarding CL and associated products. Six questions evaluated the participants' knowledge of CL and allied products, while 10 questions assessed their understanding of hygiene, CL, and accessory care and their awareness of the need to seek immediate or routine eye care for discomfort or acute complications. The accuracy of responses was compared with those provided by experienced CL practitioners (established as the Gold standard). Participants were assigned one point for each response that aligned with the experts' answers, and a score of '0' was given if the response did not match. The maximum attainable score for knowledge-related questions was 14, with scores above 10 categorized as high knowledge and scores below 10 considered low knowledge. Similarly, for practice-related questions, the maximum score was 10, and scores of seven or more were characterized as good practice, while scores below seven were considered poor practice.

Regarding the sample size, we considered a previous study on Saudi participants who used CL in which the level of knowledge was excellent in 279 (54.7%) (95% CI 50.4-59). The knowledge about CL was self-perceived in 312 (61.2%) of participants, and 40% of the participants overestimated their knowledge level regarding CL [11]. In this study, the sample size was estimated to be 779 via online calculator, statcal, but in the end, 1079 Saudi males and females aged ≥ 18 were recruited. 

Both descriptive and inferential statistical analysis of the data was carried out. Simple frequencies and percentages of the sociodemographic characteristics of the CL users, patterns, knowledge, and other categorical variables were calculated and tabulated. Chi-Square and Fisher's Exact Test are used to find the significant association between knowledge level and practice pattern with different features. Statistical significance was established at a p-value of 0.05 or less with a 95%CI. All the statistical calculations were performed using IBM SPSS Statistics for Windows, Version 29.0. (Released 2022; IBM Corp., Armonk, New York, United States).

## Results

In our study, a total of 1,079 individuals who used CL were included. The majority were female (86.4%, n=932) and aged 21-40 (65.7%, n=709) (Table 1). Most were single (53.6%, n=578), residing in cities (83.8%, n =905), and belonged to the Central (19.0%, n=205) or Eastern (27.3%, n=294) regions. Common occupations include students (42.4%, n=457) and employed individuals (29.3%, n=316). The educational level varied, with 62.1% (n=670) having a bachelor's degree. In terms of income, 48.4% (n=522) earned 5000-15000 SAR monthly. Regarding ophthalmologist visits, 69.1% (n=746) visited when needed.

**Table 1:** Sociodemographic Features of Participants

Variable		Frequency (n=1079)	Percent
Gender	Female	932	86.4
	Male	147	13.6
Age	< 20 Years	176	16.3
	21-40 Years	709	65.7
	41-60 Years	191	17.7
	> 60 Years	3	.3
Marital Status	Single	578	53.6
	Married	471	43.7
	Divorced	19	1.8
	Widowed	11	1.0
Place of Residence	Village	175	16.2
	City	904	83.8
Region	Central Region	205	19.0
	Eastern Region	295	27.3
	Northern Region	155	14.4
	Southern Region	131	12.1
	Western Region	293	27.2
Occupation	Student	457	42.4
	Employed	316	29.3
	Unemployed	94	8.7
	Housewife	168	15.6
	Retired	44	4.1
Educational Level	Primary School	6	.6
	Intermediate School	13	1.2
	High School	265	24.6
	Bachelors	670	62.1
	Post. Graduate	31	2.9
	Diploma	93	8.6
Monthly Income	< 5000 SAR	198	18.4
	5000-15000 SAR	522	48.4
	> 15000 SAR	359	33.3
Frequency of Visiting to Ophthalmologist	Never	173	16.0
	Regularly	92	8.5
	Irregularly	68	6.3
	When needed	746	69.1

The primary purpose for usage was cosmetic (36.1%, n=390) or medical (28.7%, n=310), with some using them for both purposes (35.1%, n=379) (Table 2). Usage duration varied from <1 year (28.0%, n= 302), 1-4 years (31.6%, n=341), to >5 years (40.4%, n=436). Regarding how often they wear the CL, 61.4% (n=663) said they used them part-time. A total of 616 (57.1%) did not know the type of CL they used. Among known types, soft lenses were the most common (34.4%, n=372), followed by semi-soft (6.3%, n=68), and hard (2.2%, n=24) lenses. Information sources include the internet (22.8%, n=246), optometrists (17.5%, n=189), vendors (16.7%, n=180), doctors (14.8%, n=160), family members (10.4%, n=112), friends (8.4%, n=91), and self-research (8.6%, n=93).

**Table 2:** Characteristics of Contact Lenses Used by Participants

Variable		Frequency (n=1079)	Percent
Purpose of Use of Contact Lens	Cosmetic	390	36.1
	Medical	310	28.7
	Both	379	35.1
When Do You Start Contact Lens	< 1 Year	302	28.0
	1-4 Years	341	31.6
	> 5 Years	436	40.4
How Often Do You Wear Your Contact Lenses	Not Using Now	319	29.6
	Part Time	663	61.4
	All Time	97	9.0
Type of Contact Lens	Don't Know	616	57.1
	Soft	371	34.4
	Semi-Soft	68	6.3
	Hard	24	2.2
Source of Information About Contact Lens	Internet	246	22.8
	Optometrist	189	17.5
	Vendor	180	16.7
	Doctor	160	14.8
	Family Member	112	10.4
	Friends	91	8.4
	Self	93	8.6
	Brochure	8	.7

Table 3 shows the different aspects of CL users' practices. The majority acquired lenses through a prescription (86.4%, n=932) and practiced hand hygiene (89.6%, n=967) and lens container hygiene (88.6%, n=956) before usage, and 26.3% (n=284) irregularly changed their CL. Many had attempted to learn proper usage (53.2%, n=574) and removed lenses before sleeping (94.6%, n=1021) and swimming (87.0%, n=939).

**Table 3:** Different Characteristics of Contact Lens Users

Variable		Frequency (n=1079)	Percent
General Practice & Care of Contact Lenses Usage			
Get Contact Lens Through Prescription	Yes	386	86.4
Trying to Learn the Art of Using Contact Lens	Yes	574	53.2
	Partially	359	33.3
Take Care of Hand Hygiene Before Wearing Lens	Yes	967	89.6
Take Care of Lens Container Hygiene Before Wearing Lens	Yes	956	88.6
Ever Stopped Using Contact Lens	Yes	818	75.8
Remove Contact Lens Before Sleeping	Yes	1021	94.6
Remove Contact Lens Before Swimming	Yes	939	87.0
Use Contact Lens Beyond Expiry Date	Yes	365	33.8
How do you Take Care of You Contact Lens	With Given Solution	955	88.5
	With Water	56	5.2
Anyone Else Uses Your Contact Lens	Yes	170	15.8
Complications of Contact Lens Usage			
Facial Complication Due to Usage of Lens	Yes	284	26.3
Suffer from Visual Problems (Redness, Itching) Due to Contact Lens	Yes	613	56.8
Suffer from Allergic Reactions (Runny Nose, Sneezing) Due to Wearing Lens	Yes	380	35.2
What Actions Are Taken When Allergic Reaction Occurs	Home Remedies	282	26.1
	Visit Ophthalmologist	299	27.7
Perceptions About Contact Lens Usage			
Which is Better to Use for Medical Purpose	Contact lenses	246	22.8
	Eyeglasses	616	57.1
Cosmetic Lens is More Dangerous than Medical Lens	Yes	440	40.8
It is Important to be Careful While Using Contact Lenses	Yes	965	89.4
It is Recommended to Change Contact Lenses Frequently	Yes	941	87.2
Most People Have Sufficient Knowledge About the Proper Use of Contact Lenses	Yes	202	18.7
Psychological Background Behind Lens Usage			
Is There a Psychological Factor That Lead to Contact Lens Misuse	Yes	166	15.4
Ever Diagnosed with Psychological Disease	Yes	109	10.1
Is Wearing Contact Lens for Extended Period a Sign of Insecurity	Yes	175	16.2

**Figure 1:**
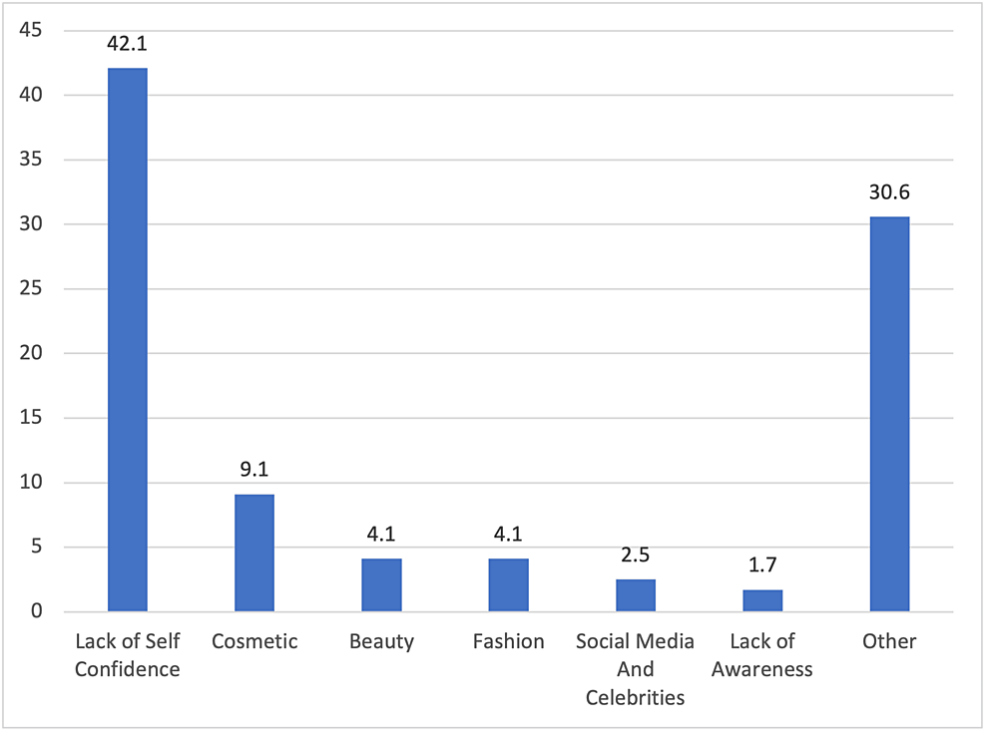
Reason for Contact Lens Use

**Figure 2:**
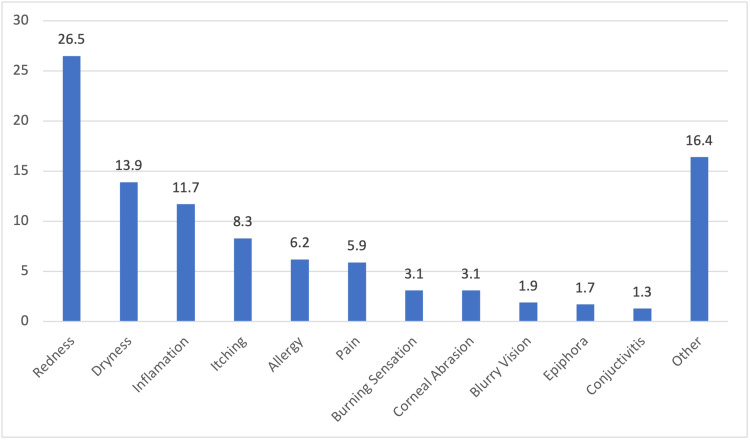
Complications Related to Contact Lens Use

**Figure 3:**
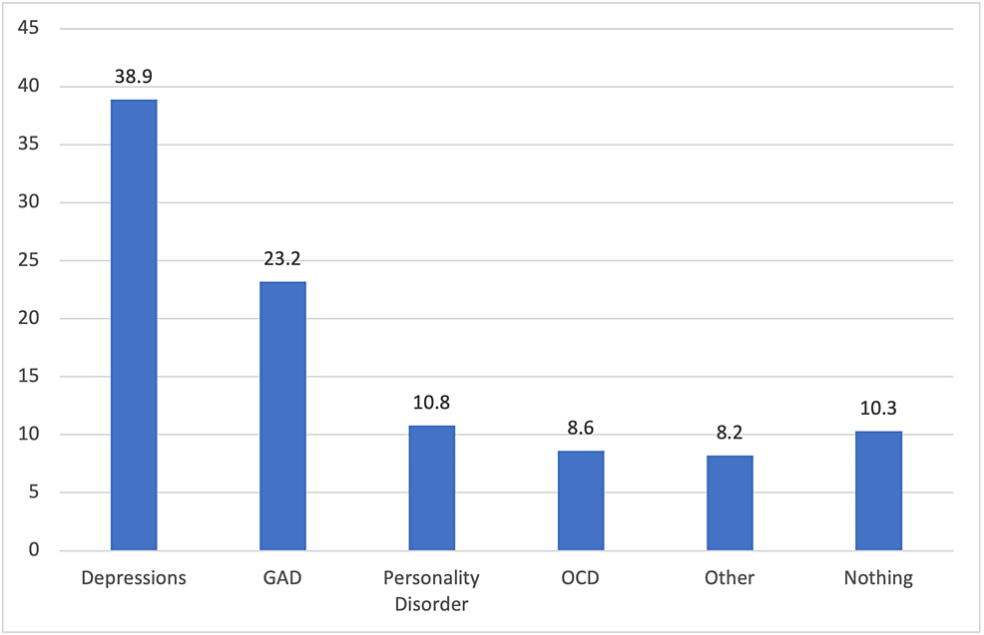
Psychological Disorders in Participants GAD: generalized anxiety disorder; OCD: obsessive-compulsive disorder

The reason for the use of CL is shown in Figure 1. However, complications arise, with 26.3% (n=284) experiencing issues due to lens usage, 56.8% (n=613) facing visual problems, and 45.8% (n=494) sometimes feeling discomfort with CL usage. Allergic reactions were reported by 35.2% (n=380), with 27.7% (n=299) seeking an ophthalmologist's help. The different complications are shown in Figure 2. The perception of lens usage indicates that eyeglasses are preferred for medical purposes (57.1%, n=616), while 40.8% (n=441) believed CL were riskier. Most emphasized cautious lens use (89.4%, n=963) and changing lenses frequently (87.2%, n=940). However, only 18.7% (n=202) believed that the population was knowledgeable about proper usage. Additionally, psychological factors influencing misuse (15.4%, n=166) and extended wear as a sign of insecurity (16.2%, n=174) highlight the complex nature of CL practices and attitudes. The different psychological disorders are shown in Figure 3.

Table 4 explores the association between knowledge levels about CL usage and various sociodemographic, complications, and psychological factors among participants. Notable findings include higher knowledge among participants aged 21-40 years (p = 0.009), those who visit ophthalmologists irregularly (p = 0.032), and those who use lenses for cosmetic purposes (p = 0.027). Lower knowledge is linked to complications (p < 0.001). No significant gender or educational status differences are observed. The study suggests that age, purpose of use, frequency of ophthalmologist visits, and complications might influence contact lens knowledge. However, other factors like gender, educational status, and psychological disorder prevalence do not show significant associations.

**Table 4:** Association of Level of Knowledge about Contact Lens Use with Sociodemographic and Psychological Backgrounds and Complications. *Statistically significant (p< 0.05, p<0.001). Data presented as frequency (n)

Variable		Knowledge Level about Usage of Contact Lenses			Sig. Value
		Low Knowledge	High Knowledge		
Gender	Female	178	754	0.341	
	Male	33	114		
Age	< 20 Years	48	128	0.009	
	21-40 Years	119	590		
	41-60 Years	43	148		
	> 60 Years	1	2		
Residence	Village	40	135	0.229	
	City	171	733		
Regions	Central Region	41	164	0.066	
	Eastern Region	72	223		
	Northern Region	23	132		
	Southern Region	19	112		
	Western Region	56	237		
Educational Status	Student	99	358	0.565	
	Employed	58	258		
	Unemployed	14	80		
	Housewife	32	136		
	Retired	8	36		
Educational Level	Primary School	2	4	0.299	
	Intermediate School	0	13		
	High School	55	210		
	Bachelors	128	542		
	Post. Graduate	4	27		
	Diploma	22	71		
Marital Status	Single	119	459	0.678	
	Married	85	386		
	Divorced	4	15		
	Widowed	3	8		
Monthly Income	< 5000 SAR	35	163	0.705	
	5000-15000 SAR	102	420		
	> 15000 SAR	74	285		
Frequency Of Visiting Ophthalmologist	Never	42	131	0.032	
	Regularly	12	80		
	Irregularly	7	61		
	When needed	150	596		
Purpose of Using Contact Lens	Cosmetic	65	325	0.027	
	Medical	76	234		
	Both	70	309		
Complications with Contact Lens	No	192	603	<0.001	
	Yes	19	265		
Psychological Disorder in Saudi Populations	No	184	717	0.172	
	Yes	19	90		

Table 5 shows the association between practice patterns of contact lens usage and various sociodemographic, complications, and psychological factors among participants. Notable findings include better practice patterns among those living in cities (p = 0.049) and higher monthly income brackets (p = 0.002). Better practices are linked to cosmetic use (p < 0.001) and fewer complications (p < 0.001). Practice patterns do not significantly differ based on gender, age, region, educational status, marital status, frequency of ophthalmologist visits, or psychological disorders. The study suggests that residence, income, purpose of use, and complications might influence contact lens practice patterns, highlighting potential areas for educational interventions.

**Table 5:** Association of Practice Pattern of Contact Lens Use with Sociodemographic and Psychological Backgrounds and Complications. *Statistically significant (p< 0.05, p<0.001). Data presented as frequency (n)

		Practice Pattern Regarding Contact Lenses Use		Sig. Value
		Poor Practice Pattern	Better Practice Pattern	
Gender	Female	143	789	0.078
	Male	31	116	
Age	< 20 Years	36	140	0.255
	21-40 Years	105	604	
	41-60 Years	32	159	
	> 60 Years	1	2	
Residence	Village	37	138	0.049
	City	137	767	
Regions	Central Region	32	173	0.519
	Eastern Region	46	249	
	Northern Region	22	133	
	Southern Region	28	103	
	Western Region	46	247	
Educational Status	Student	82	375	0.339
	Employed	54	262	
	Unemployed	11	83	
	Housewife	21	147	
	Retired	6	38	
Educational Level	Primary School	2	4	0.564
	Intermediate School	2	11	
	High School	44	221	
	Bachelors	101	569	
	Post. Graduate	5	26	
	Diploma	20	73	
Marital Status	Single	101	477	0.554
	Married	65	406	
	Divorced	5	14	
	Widowed	3	8	
Monthly Income	< 5000 SAR	31	167	0.002
	5000-15000 SAR	79	443	
	> 15000 SAR	64	295	
Frequency Of Visiting Ophthalmologist	Never	43	130	0.154
	Regularly	9	83	
	Irregularly	6	62	
	When needed	116	630	
Purpose of Using Contact Lens	Cosmetic	61	329	<0.001
	Medical	60	250	
	Both	53	326	
Complications with Contact Lens	No	165	630	<0.001
	Yes	9	275	
Psychological Disorder in Saudi Populations	No	147	754	0.907
	Yes	16	93	

## Discussion

CLs are frequently utilized to correct refractive errors and for cosmetic reasons. Individuals with refractive errors can substitute eyeglasses with CLs to enhance their quality of life and provide greater flexibility during activities, which is preferable for younger age groups [13,14]. Studies indicate CL complications are more common in adolescents and young adults than older adults [15]. Improper care and poor compliance with professional advice can lead to sight-threatening eye complications for CL users [16,17]. 

The current study aimed to assess the use, misuse, and complications of CL among the general population in Saudi Arabia. As for CL use, the study showed that about one-third of the study participants used CL for cosmetic purposes. In contrast, a lesser percentage used them for medical reasons, and others used them for both purposes. Also, two-thirds used CL part-time, which is consistent with cosmetic use purposes. Most CL users did not know what type they use, which is unsafe behavior. In Saudi Arabia, Alzahrani et al. documented that most people who use CL do not have any vision issues [18]. Non-users were found to believe that using CL could be harmful more often (12.4%) than those who use them (2.93%). The most common reason for using CL was to imitate others. In another study conducted by Abahussin et al. among Saudi university students, a similar segment of CL users applied them for cosmetic purposes [19]. Globally, the prevalence of CL use among university students is 19.8%, and most CL users (82.15%) were female. According to a study by Zhu et al., aesthetics was reported as the main reason for CL use (57.91%), while comfort was identified as the top consideration when purchasing CLs (75.76%) [20].

As for practice, the current study showed that most users got CL by prescription. As for CL care, most users removed CLs before sleeping, took care of hand hygiene before wearing lenses, and took care of lens container hygiene before wearing lenses. On the other hand, about three-fourths of the users wear CL either part-time or all the time, while one-third of CL users admitted to using their CL beyond the expiry date. Tajunisah et al. found that the duration of CL wear varied from less than six months to two years or more, 53 (43.8%) cited cosmetic purposes for wearing CL, and 16 (13.2%) did not remove their CL before sleeping at night [21]. Ahmad Najmee et al. reported that 88% of the students surveyed reported following hand hygiene before handling CLs [22].

Additionally, Ahmad Najmee et al. reported that 85.7% of students, in their study, strictly adhered to the cleaning instructions for their CL [22]. Also, most students (55.6%) reported wearing CL less than eight hours a day. Furthermore, two students (3.2%) admitted that they occasionally did not remove their CL before sleeping at night. Most students (58, 92.1%) never slept with their CLs on at night. In contrast, in Saudi Arabia, Alobaidan et al. found that two-thirds of CL users had procured CL without a prescription [11]. Only half of the participants were confident about their skills in using CL, one in five CL users was irregular in changing their CL, and hand hygiene was good in three-fourths of participants. Another study revealed that 2.6% of participants changed sterile solution daily, 15.8% changed the lens box monthly, 81.2% washed their hands before, 89.6% washed their lenses before and 33.2% after wearing the lenses, and 37.2% followed the correct washing method which is similar to the current study findings [23]. 

As for complications associated with CL use, the current study showed that the most experienced complications were eye problems (redness and itching), allergic reactions (runny nose, sneezing) due to wearing lenses, and facial complications. Based on the literature review, the prevalence of CL-related complications ranges between 23% and 94% [24-27]. Kim et al. reported corneal erosion, sterile corneal infiltrate, allergic disease, conjunctival injection, corneal ulcer, and dry eye syndrome [28]. In China, Li et al. documented that dry eye (36.8%), followed by superficial punctuate keratitis (36.1%) with blepharitis and meibomian gland dysfunction (MGD) were the most reported CL use complications [8]. In Saudi Arabia, it was found that about one-fifth of CL users presented with laboratory-proven infectious keratitis [16]. Also, Alzahrani et al. found that dryness was a well-known complication in current users (P < 0.05), and evening discomfort was a well-known one in previous users [18].

Considering knowledge about CL, the current study showed that most of the participants (more than three-fourths) had good knowledge. Higher knowledge was significantly detected among older users, higher frequency of visiting ophthalmologists, using CL for cosmetic purposes, and having complications unrelated to other sociodemographic characteristics. Alobaidan et al. showed similar knowledge levels, where 54.7% of CL users had excellent knowledge levels [11]. Boqursain et al. found that the total mean knowledge score for CL was 30.1 ± 7.74, higher in females (31.5 ± 7.09) than in males [29]. The current study showed that the internet, optometrists, vendors, and doctors were the most reported sources of information about the use of CL.

The study has several limitations that should be considered when interpreting the findings. Firstly, the use of a snowball convenience sampling technique introduces potential bias in the sample, as the initially recruited participants may have specific characteristics or affiliations that may not be representative of the general population. This limits the generalizability of the findings to a broader population. Secondly, the reliance on a self-administered questionnaire for data collection introduces the possibility of response bias, as participants may provide inaccurate or incomplete information. The varying understanding and interpretation of the questions could also impact the accuracy of the collected data. Moreover, the absence of biological sample collection restricts the assessment of certain variables or outcomes that may be better examined through biological measures, thereby limiting the depth of analysis and understanding. Additionally, the online nature of data collection may introduce limitations in ensuring the validity and reliability of responses, as researchers have no control over participants' environments or potential distractions. The exclusion of individuals without internet access or familiarity with online survey platforms may also introduce selection bias. Lastly, while a pilot study will be conducted, it may not capture all potential issues or limitations of the questionnaire, which could impact the accuracy or validity of the collected data.

## Conclusions

This study highlights the complex nature of CL use in the general population of the Kingdom of Saudi Arabia. While a significant percentage of participants use contact lenses for cosmetic purposes, there is a lack of awareness regarding the lenses being used. Despite efforts to promote proper practices such as obtaining lenses through a prescription and practicing good hygiene, many participants reported experiencing complications and discomfort associated with contact lens use. Additionally, the study found that age, purpose of use, frequency of ophthalmologist visits, residence, and income are factors that may influence contact lens knowledge and usage patterns.

Based on these findings, it is crucial to increase awareness and education regarding proper contact lens usage, particularly among those using lenses for cosmetic purposes. Targeted interventions should be developed to address complications, enhance knowledge, and promote better practices, taking into account sociodemographic factors such as age, purpose of use, residence, and income. By addressing these issues, we can work towards improving CL safety and promoting better eye health in the population.
